# An Analytical Approach to Flow-Guided Nanocommunication Networks

**DOI:** 10.3390/s20051332

**Published:** 2020-02-29

**Authors:** Rafael Asorey-Cacheda, Sebastian Canovas-Carrasco, Antonio-Javier Garcia-Sanchez, Joan Garcia-Haro

**Affiliations:** Department of Information and Communication Technologies, Universidad Politécnica de Cartagena, 30202 Cartagena, Spain; sebas.canovas@upct.es (S.C.-C.); antoniojavier.garcia@upct.es (A.-J.G.-S.); joang.haro@upct.es (J.G.-H.)

**Keywords:** flow-guided nano-networks, analytical model, nanocommunications

## Abstract

Continuous progress of nanocommunications and nano-networking is opening the door to the development of innovative yet unimaginable services, with a special focus on medical applications. Among several nano-network topologies, flow-guided nanocommunication networks have recently emerged as a promising solution to monitoring, gathering information, and data communication inside the human body. In particular, flow-guided nano-networks display a number of specific characteristics, such as the type of nodes comprising the network or the ability of a nano-node to transmit successfully, which significantly differentiates them from other types of networks, both at the nano and larger scales. This paper presents the first analytical study on the behavior of these networks, with the objective of evaluating their metrics mathematically. To this end, a theoretical framework of the flow-guided nano-networks is developed and an analytical model derived. The main results reveal that, due to frame collisions, there is an optimal number of nano-nodes for any flow-guided network, which, as a consequence, limits the maximum achievable throughput. Finally, the analytical results obtained are validated through simulations and are further discussed.

## 1. Introduction

Recent advances in nanotechnology are laying the foundations for the development of novel medical applications that can significantly improve the effectiveness of disease diagnosis and treatment [1,2,3,4,5]. One of the most promising techniques is based on the deployment of size-constrained devices endowed with a wireless communication module, known as nano-nodes, inside the human body, where larger devices would be inappropriate or excessively invasive. These nano-nodes, measured in micrometers, could be injected into the bloodstream providing real time in-vivo measurements of different elements present in the blood. For example, each nano-node could be equipped with a nano-sensor similar to that proposed in [6], enabling the detection of cancer biomarkers at an early stage. This type of nano-network, in which nano-nodes continuously move within a well-defined flow, is known as a flow-guided nanocommunication network [7].

Flow-guided nanocommunication networks pose two important challenges that must be addressed. On the one hand, as the miniaturization of the radiating antenna integrated into each nano-node demands the use of high frequencies to communicate (around 1 THz [2,8]), the attenuation of electromagnetic signals within the human body (largely made up of water) is very high [9,10]. Inherently, this high path loss severely limits the communication range, making direct communication between nano-nodes and a macro device (e.g., a wearable device or a smartphone) impractical [3,11]. On the other hand, the extremely limited amount of energy that can be stored in a nano-node (due to technological constraints [12]) requires the use of a piezoelectric nano-generator to recharge the battery periodically [13,14]. Therefore, the energy balance in flow-guided nano-networks must be examined considering both the energy consumed and the energy harvested from the environment.

To alleviate these two shortcomings caused by the extremely reduced size of nano-nodes, larger and less resource-constrained devices called nano-routers act as a middle network tier that gathers all the raw data generated by nano-nodes, and then sends them to a macro device, which can connect the nano-network to the Internet [15,16,17,18]. Even though nano-routers are larger in size than nano-nodes, they are envisaged to be small enough to be implanted in the human body. Thus, through strategic placement, the distance of the communication link between the nano-node and nano-router can be minimized to ensure that nano-nodes are able to transmit the generated data, employing small amounts of available energy [11]. Thereby, nano-nodes will successfully transmit a data frame when they pass through the coverage range of a nano-router. As nano-nodes must keep energy consumption as low as possible, we consider that a nano-node ignores whether it is in the coverage area or not; i.e., no energy is consumed listening to the channel.

As the characteristics of flow-guided nano-networks are quite different to other nano-network topologies, a theoretical framework capturing all their peculiarities is needed to properly model their behavior and set theoretical boundaries on their performance. For this purpose, in this paper, we propose a general analytical model to mathematically evaluate different metrics of flow-guided nano-networks. We then derive closed-form equations for the maximum achievable throughput as a function of the number of nano-nodes in the network, the coverage range of the nano-routers, the battery recharge frequency, the average nano-node velocity, and the number of transmission slots. We also employ these equations to analyze the impact on the throughput of each one of these parameters. To summarize, the main contributions of this work are as follows:

- We develop an analytical model capturing all the particularities of flow-guided networks. We note that this model is general enough to be implemented in different applications in which flow-guided networks could be used. To the best of our knowledge, no previous mathematical model for this type of networks exists.
- We provide insightful results about the achievable throughput of flow-guided nano-networks as a function of different important parameters.
- We calculate the maximum number of nano-nodes that can compose a flow-guided nano-network without penalizing the total throughput of the network. It should be noted that, when the number of nano-node is excessively high, data frame collisions significantly hinder the throughput achieved.
- We assume a time-slotted transmission technique to increase the achievable throughput of flow-guided nano-networks. Results obtained show that this slotted technique substantially increases the total capacity of the network.
- We validate the proposed mathematical framework by simulating a flow-guided nano-network under different scenarios. The results obtained with the simulations have been compared to those attained by the analytical equations, showing that the mathematical framework accurately models the behavior of a flow-guided nano-network.

The rest of the paper is organized as follows. Section 2 outlines all the related work. Section 3 rigorously defines the analytical model. All the results are discussed in Section 4, including those obtained from simulations. The impact on the network throughput of each model parameter is discussed, highlighting the benefits of employing a slotted transmission scheme and the effect of frame length on overall performance. Finally, Section 5 concludes the paper. Appendix A further discusses the behavior of the simultaneous number of collisions in flow-guided nano-networks.

## 2. Related Work

Since nano-networks are still in their infancy, many works in the literature are devoted to theoretically modeling the behavior of different types of nano-networks. Authors in [19] proposed a routing framework to maximize the overall network throughput of a hierarchical nano-network. The network architecture studied in that work consisted of static nano-sensors and nano-controllers, in which nano-sensors were divided into clusters, each one associated to a nano-controller. In contrast, our work considers mobile nano-nodes and static nano-routers. In [3], a theoretical study of the communication capabilities of a nano-network deployed inside a human body is developed. By thoroughly taking into account the dielectric properties of different biological tissues, the maximum channel capacity as a function of the transmission distance for different communication schemes is obtained. Results suggest that, since the transmission distances are limited to obtain an acceptable capacity, the development of nano-networks in the human body requires multi-hop network topologies (as proposed in our work).

In [20], the maximum achievable throughput of a nano-network composed of *n* randomly distributed identical nodes is analyzed. The main characteristics of an electromagnetic nano-network are adequately considered and captured in the theoretical model. Results reveal that, for high-density nano-networks (over $10^{7}$ nodes per unit area), interferences between nano-nodes limit the achievable throughput. Similarly, Section 3 of our work determines the maximum achievable throughput and the number of nano-nodes necessary to obtain it particularized to flow-guided nano-networks.

Regarding the study of flow-guided nano-networks, the work in [11] proposed a realistic flow-guided nano-network, following a hierarchical topology based on three network layers: nano-nodes, a nano-router, and an Internet gateway. It was designed to monitor the human body, with the nano-nodes injected into the bloodstream communicating with a nano-router strategically implanted in the skin. Nano-nodes transmit the generated data to this static nano-router when they are in its vicinity. In turn, the nano-router sends the received data to an Internet gateway placed outside the body, connecting the nano-network to the Internet. However, the main limitation of this flow-guided nano-network is that only one nano-router is considered, severely restricting the nano-network capacity. It should be noted that this work proposes a deterministic and realistic case of using flow-guided nano-networks. Conversely, our present work provides a general mathematical model to analyze the different performance metrics of the network.

Finally, the authors in [7] proposed a mathematical framework to derive optimal transmission policies for flow-guided nano-networks. Results indicated that intelligently using the limited resources of nano-nodes, the network throughput can be increased in a realistic case of study (based on the human circulatory system). This work laid the foundations to formulate and derive our analytical model as described in the following sections.

## 3. Analytical Model

### 3.1. Model Description

**Figure 1:**
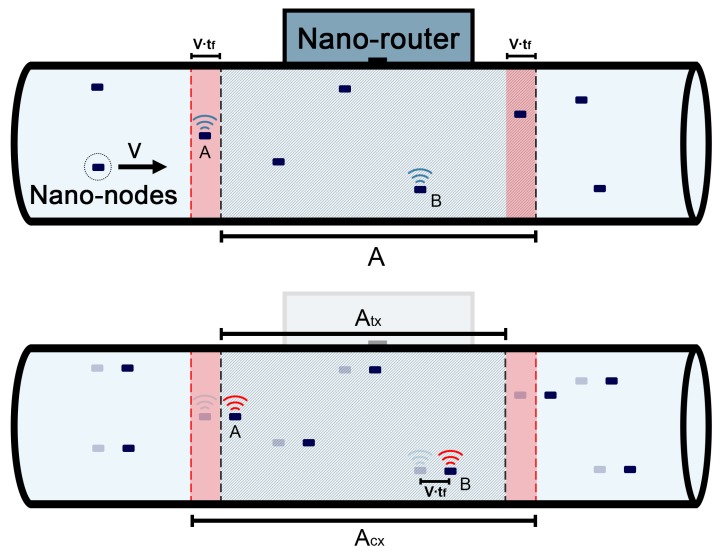
Scenario in which transmissions from Nano-Nodes A and B collide. The upper part of the figure represents the initial state at $t_{0}$ (Nano-Node A within the transmission zone and Nano-Node B within the collision zone; i.e., transmissions do not collide yet). The lower part of the figure represents the final state after the transmissions end at $t_{0}+t_{f}$ (Nano-Nodes A and B within the transmission zone; i.e., a collision occurs).

The analytical model is based on the following assumptions:

- There are *n* nano-nodes uniformly distributed along the flow, n≥1, n∈N.
- The flow network is a closed circuit in which the nano-nodes continuously circulate. An example of a flow network might be the blood circulatory system of the human body.
- A nano-node moves within the flow at average speed *v* and requires *T* time units to complete a round.
- A nano-node battery is charged every 1/f time units. Moreover, due to energy constraints, a nano-node can only transmit one data frame per battery charge. The work in [14] provides more details on a realistic model based on piezo-electric elements to charge a nano-node battery. In this paper, we assume a similar battery charging mechanism. Thus, a nano-node can only harvest enough energy to transmit a single frame every 1/f time units.
- A successful transmission can only occur when a nano-node is close enough to a nano-router and no other nano-node transmits at the same time (see Figure 1). For the sake of simplicity, without a loss of generality, let us assume that there is a region in the flow of length *A*, in which a nano-node frame transmission can successfully reach a network nano-router (a nano-node is under the nano-router coverage zone). Thus, the probability of a nano-node being in the coverage zone ($p_{A}$) can be modeled as
  (1) $$p_{A}=\frac{A}{vT}$$
  For instance, nano-router coverage is a volume that could be a section of a vein or an artery; its characterization as the length of magnitude *A* does not imply a loss of generality. Let us assume that the flow network is cylindrically shaped and denote *r* as its radius. Thus, the volume of the area of coverage is $A\pi r^{2}$, and the total network volume is $vT\pi r^{2}$. As a consequence, $A\pi r^{2}/\left(vT\pi r^{2}\right)=A/\left(vT\right)=p_{A}$. The nano-node coverage model of this paper is based on [11]. It can be consulted for more details on nano-node coverage issues in flow guided nano-networks.
- Let us assume that A/v<1/f, i.e., the time between transmissions is longer than the time used by a nano-node to cross the coverage area. In other words, a nano-node cannot perform more than one transmission when crossing the coverage area.
- A nano-node ignores if it is within the coverage area due to its energy limitations. Thus, any nano-node will try to perform a successful transmission at every battery charge cycle, independently of its position in the flow-guided network. These transmissions can occur at any moment between two consecutive battery charges.
- The model described in this paper assumes that the time between two battery charges, 1/f, is divided into σ slots (σ≥1, σ∈N) and a nano-node transmits a frame randomly, with a probability 1/σ, in one of these slots. The maximum number of slots is limited by the frame size. Thus, if $t_{f}$ is the time required to transmit a frame, $t_{f}\leq {\left(f\sigma \right)}^{-1}$.
- A successful frame transmission takes place if it starts and ends within the coverage area and no collision occurs. As a consequence, the transmission zone, $A_{tx}$, is smaller than the coverage area, $A_{tx}=A-vt_{f}$, as any successful transmission must start and end within the coverage area (see Figure 1). Thus, note that the probability of a nano-node being in the transmission zone is as follows:
  (2) $$p_{tx}=\frac{A-vt_{f}}{vT},\;vt_{f}<A$$
- A collision occurs if one or more transmissions start or end within the coverage zone while another transmission in the transmission zone takes place. As a consequence, the collision zone, $A_{cx}$, is larger than the coverage area, $A_{cx}=A+vt_{f}$, as a collision can only be avoided if it starts and ends outside the coverage area (see Figure 1). Thus, the probability of a nano-node being in the collision zone is as follows:
  (3) $$p_{cx}=\frac{A+vt_{f}}{vT},\;vt_{f}<A$$
- For the sake of simplicity, in many cases, it can be assumed that $vt_{f}⋘A$, which is a realistic assumption according to the values used in [7]. Thus, a frame collision event can only be considered when two or more nodes transmit within the coverage area of the nano-router in the same transmission slot. As a consequence, transmissions starting outside the coverage area and ending inside, or vice versa, can be neglected. If $vt_{f}⋘A$ holds, the following approximation can be made:
  (4) $$p_{tx}\approx p_{cx}\approx p_{A},\;vt_{f}⋘A$$
- This model assumes that A≪vT, as in [7], which is a realistic assumption in flow-guided nano-networks.

Table 1 specifies all the variables used in this analytical model.

### 3.2. Achievable Throughput

As a first approach, let us assume that $A\gg vt_{f}$ and $p_{tx}\approx p_{cx}\approx p_{A}$; i.e., frame length can be neglected. At the end of this section, throughput equations will be generalized for arbitrary lengths of $t_{f}$.

According to the the model of Section 3.1, it is possible to obtain an analytical equation of the achievable network throughput. Thus, a successful transmission happens when a nano-node transmits a frame within a nano-router coverage area and no other nano-node is in the coverage area or, if it is within, it does not transmit in the same time slot.

As nano-nodes are uniformly distributed along the flow network, the probability of transmitting within the coverage area depends on (i) $p_{A}$ and (ii) the probability of performing a transmission while crossing the coverage area. The probability of a nano-node being outside of the coverage area can be derived from Equation (1) as $1-p_{A}$. Moreover, the probability of another nano-node being in the coverage area and not transmitting in a given time slot, $p_{A,ntx}$, can be modeled as follows:

(5) $$p_{A,ntx}=p_{A}(1-\frac{1}{\sigma })=\frac{A}{vT}(1-\frac{1}{\sigma })$$

The successful transmission probability to a nano-router for a node, $p_{A,tx}$, can be formulated as follows:

(6) pA,tx=pA1σ∑k=0n−1(n−1k)(1−pA)n−1−kpA,ntxk

Equation (6) represents the probability of a nano-node within the coverage area transmitting a frame without collisions. The summation of Equation (6) can be simplified as follows:

(7) $$p_{A,tx}=\frac{p_{A}}{\sigma }{\left(1-\frac{p_{A}}{\sigma }\right)}^{n-1}=\frac{A}{vT\sigma }{\left(1-\frac{A}{vT\sigma }\right)}^{n-1}$$

From Equation (7), the network throughput can be derived as $\mathrm{Th}\left(n,A,\sigma \right)=nf\sigma p_{A,tx}$, corresponding to *n* nano-nodes and the the event transmission rate, fσ:

(8) $$\mathrm{Th}\left(n,A,\sigma \right)=\frac{nAf}{vT}{\left(1-\frac{A}{vT\sigma }\right)}^{n-1}$$

Equation (8) shows that network throughput increases with the number of slots, as can be expected, as well as for a moderate number of nano-nodes in the network. However, if the number of nano-nodes is too large, throughput converges to 0.

#### 3.2.1. Maximum Achievable Throughput

The number of nano-nodes, $n_{th}$, maximizing Equation (8), can be easily obtained from dTh(n,A,σ)/dn=0:

(9) $$\frac{d\mathrm{Th}(n,A,\sigma )}{dn}=\frac{Af}{vT}{\left(1-\frac{A}{vT\sigma }\right)}^{n-1}(1+n\log(1-\frac{A}{vT\sigma }))=0$$

Thus, $n_{th,\sigma }$ is expressed as follows:

(10) $$n_{th,\sigma }=-\log{\left(1-\frac{A}{vT\sigma }\right)}^{-1}$$

This model assumes that A≪vTσ, and Equation (10) can therefore be approximated by the following:

(11) $$n_{th,\sigma }\approx \frac{vT\sigma }{A},\;A\ll vT\sigma$$

As can be observed from Equations (10) and (11), the number of nano-nodes required to achieve maximum throughput grows with the number of slots. This can be seen as a drawback, since maintaining the number of nano-nodes as low as possible might be a desired design goal. However, increasing the number of slots also raises throughput for any value of *n*, as demonstrated in Section 3.2.2. Note that, although slotted transmission mechanisms require synchronization that cannot be straightforward to implement, flow-guided nano-network synchronism is externally and naturally provided to nano-nodes by the battery charging cycles.

The maximum achievable throughput as a function of *A* and σ will be as follows:

(12) $${\mathrm{Th}}_{n}\left(A,\sigma \right)=\frac{-Af}{vT\log(1-\frac{A}{vT\sigma })}{\left(1-\frac{A}{vT\sigma }\right)}^{-\log{\left(1-\frac{A}{vT\sigma }\right)}^{-1}-1}$$

Equation (12) can be simplified as follows (*e* is Euler’s number):

(13) $${\mathrm{Th}}_{n}\left(A,\sigma \right)=\frac{-Af}{evT\log(1-\frac{A}{vT\sigma })(1-\frac{A}{vT\sigma })}$$

Since in this model it is assumed that A≪vTσ, Equation (13) can be further approximated by the following:

(14) $${\mathrm{Th}}_{n}\left(\sigma \right)\approx \frac{f\sigma }{e},\;A\ll vT\sigma$$

As a consequence, the maximum achievable throughput does not rely on *A*, but on the number of slots, σ.

#### 3.2.2. Benefits of Slot Utilization

The positive impact of using slots on the achievable throughput can be quantified as $\eta_{th}\left(A,\sigma \right)={\mathrm{Th}}_{n}\left(A,\sigma \right)/{\mathrm{Th}}_{n}\left(A,1\right)$. Using Equation (14),

(15) $$\eta_{th}\left(A,\sigma \right)\approx \sigma ,\;A\ll vT\sigma$$

However, this improvement can only be attained by increasing the number of nano-nodes in the flow-guided network. Let us define $\eta_{n}\left(A,\sigma \right)=n_{th,\sigma }/n_{th,1}$. Thus,

(16) $$\eta_{n}\left(A,\sigma \right)=\frac{\log(1-\frac{A}{vT})}{\log(1-\frac{A}{vT\sigma })}$$

Assuming that A≪vTσ, Equation (16) can be approximated by the following:

(17) $$\eta_{n}\left(A,\sigma \right)\approx \sigma ,\;A\ll vT\sigma$$

Equations (15) and (17) show that achieving any arbitrary improvement in throughput requires increasing the number of slots and therefore the number of nano-nodes proportionally. Let us define $\eta_{s}\left(n,A,\sigma \right)$ as the improvement in throughput for an arbitrary number of slots, but with the same number of nano-nodes, $\eta_{s}\left(n,A,\sigma \right)=\mathrm{Th}\left(n,A,\sigma \right)/\mathrm{Th}\left(n,A,1\right)$:

(18) $$\eta_{s}\left(n,A,\sigma \right)={\left(\frac{vT-A/\sigma }{vT-A}\right)}^{n-1}$$

If σ is large enough, σ→∞, Equation (18) can be approximated by the following:

(19) $$\eta_{s}\left(n,A,\sigma \right)\approx {\left(\frac{vT}{vT-A}\right)}^{n-1},\;\sigma \to \infty$$

Moreover, in this model, A≪vT allows us to further approximate Equation (19) as follows:

(20) $$\eta_{s}\left(n,A,\sigma \right)\approx 1+\frac{(n-1)A}{vT},\;\sigma \to \infty ,\;A\ll vT$$

Equation (19) shows that throughput improvement is bounded and depends on the number of nano-nodes. Moreover, Equation (20) imposes that n∼vT/A, in order for $\eta_{s}\left(n,A,\sigma \right)$ to be a significant value. For most scenarios, as will be discussed below in Section 4, this can be difficult to satisfy, and $\eta_{s}\left(n,A,\sigma \right)\approx 1$; i.e., if the number of nano-nodes remains constant, increasing the number of slots does not provide a substantial improvement in throughput.

#### 3.2.3. Length of the Coverage Area

Since the length *A* modeling the coverage area is relevant for the values of $p_{A}$, $p_{tx}$, and $p_{ntx}$, respectively; it can be argued that there is a value of *A* that maximizes throughput as a function of *n* and σ. However, note that the value of *A* is constrained by an upper limit, $A_{max}$, derived from the maximum transmission power of the nano-node. On the other hand, a nano-node can reduce its transmission power below its maximum and, consequently, reduce the length *A* of the coverage area:

- A larger value of *A* increases the probability of transmitting in the coverage area, $p_{tx}$, as well as the probability of additional nodes in this area, $p_{A}$, and the probability of frame collision, $p_{ntx}$.
- A lower value of *A* reduces $p_{tx}$, as well as $p_{A}$ and $p_{ntx}$.

An optimal value of *A* can be obtained from dTh(n,A,σ)/dA=0:

(21) $$\frac{d\mathrm{Th}(n,A,\sigma )}{dA}=\frac{nf}{vT}{\left(1-\frac{A}{vT\sigma }\right)}^{n-2}(1-\frac{nA}{vT\sigma })=0$$

Equation (21) has two solutions. The first one is A=vTσ, which is only valid if σ=1 because A≤vT. The second solution is A=vTσ/n, which requires n≥σ to be valid. In general, the value of *A* that maximizes throughput is as follows:

(22) $$A_{th}=\min\{A_{max},\frac{vT\sigma }{n}\}$$

Equation (22) evidences that increasing the number of nodes and decreasing the length of the coverage area leads to a higher network throughput. Moreover, from Equation (22), one can also infer that, in order to achieve maximum throughput, the following condition must hold: $n\geq vT\sigma /A_{max}$. In other words, below this threshold, it is not possible to achieve maximum throughput.

The maximum achievable throughput as a function of *n* and σ is as follows:

(23) $${\mathrm{Th}}_{A}\left(n,\sigma \right)=f\sigma {\left(1-\frac{1}{n}\right)}^{n-1}$$

If *n* is large enough, n→∞, Equation (23) can be approximated by the following:

(24) $${\mathrm{Th}}_{A}\left(n,\sigma \right)\approx \frac{f\sigma }{e},n\to \infty$$

Therefore, Equation (24) reveals that the length of the coverage area is not relevant for the maximum achievable throughput, and is also consistent with Equation (14).

#### 3.2.4. Generalization of Throughput for Arbitrary Frame Lengths

If the frame duration, $t_{f}$, cannot be neglected, equations in Section 3.2 can be generalized to introduce the frame length. Thus, the probability of transmitting within the transmission zone depends on $p_{tx}$ and on the probability of performing a transmission while crossing the transmission zone. Moreover, the probability of another nano-node being in the collision zone and not transmitting in a given time slot, $p_{A,ntx}$, can be modeled as follows:

(25) $$p_{A,ntx}=p_{cx}(1-\frac{1}{\sigma })=\frac{A+vt_{f}}{vT}(1-\frac{1}{\sigma })$$

The successful transmission probability to a nano-router, for any nano-node, $p_{A,tx}$, can then be formulated as follows:

(26) pA,tx=ptx1σ∑k=0n−1(n−1k)(1−pcx)n−1−kpA,ntxk=ptxσ(1−pcxσ)n−1=A−vtfvTσ(1−A+vtfvTσ)n−1

From Equation (26), the network throughput can be derived as $\mathrm{Th}\left(n,A,\sigma ,t_{f}\right)=nf\sigma p_{A,tx}$, corresponding to *n* nano-nodes, and the the total event transmission rate, fσ:

(27) $$\mathrm{Th}\left(n,A,\sigma ,t_{f}\right)=\frac{nf(A-vt_{f})}{vT}{\left(1-\frac{A+vt_{f}}{vT\sigma }\right)}^{n-1}$$

Equation (27) represents the frames per time unit that can reach the nano-router. However, maximum throughput does not only depend on the number of frames, but also on the duration of these frames. Thus, throughput is maximized when the transmission channel utilization reaches its maximum.

The number of nano-nodes, $n_{th}$, maximizing Equation (8), can be easily obtained from dTh(n,A,σ)/dn=0. Thus, $n_{th,\sigma }$ is as follows:

(28) $$n_{th,\sigma }=-\log{\left(1-\frac{A+vt_{f}}{vT\sigma }\right)}^{-1}\approx \frac{vT\sigma }{A+vt_{f}},\;A\ll vT\sigma$$

As can be observed from Equation (28), the number of nano-nodes required to achieve maximum throughput grows with the number of slots.

### 3.3. Collision Rate

Beyond the analysis of the network throughput in flow-guided nano-networks, it is also worth studying frame collision behavior.

This analysis is quite straightforward, since a collision occurs when two or more nano-nodes transmit at the same time slot within the coverage area. This probability, $p_{col}$, can be modeled as follows:

(29) pcol=∑k=2n(nk)pAk(1−pA)n−k(∑m=2k(km)1σm(1−1σ)k−m)

Equation (29) determines the probability of two or more nano-nodes being within the coverage area transmitting simultaneously in the same time slot. Moreover, Equation (29) can be simplified as follows:

(30) $$p_{col}=1-{\left(1-\frac{A}{vT\sigma }\right)}^{n-1}(1+\frac{(n-1)A}{vT\sigma })$$

Let us define $\mathrm{Col}\left(n,A,\sigma \right)=f\sigma p_{col}$ as the number of collisions per unit of time. That is, a collision might occur with a frequency fσ:

(31) $$\mathrm{Col}\left(n,A,\sigma \right)=f\sigma (1-{\left(1-\frac{A}{vT\sigma }\right)}^{n-1}(1+\frac{(n-1)A}{vT\sigma }))$$

Demonstrating that $\lim_{n\to \infty }p_{col}=1$ is straightforward; i.e., if there are too many nano-nodes in the flow network, all transmissions within the coverage area will collide.

### 3.4. Channel Utilization and Frame Length

Regarding channel utilization, $U(n,A,\sigma ,t_{f})$, it can be defined as the number of frames per time unit times the frame length (throughput is taken from Equation (27)):

(32) $$U\left(n,A,\sigma ,t_{f}\right)=\mathrm{Th}\left(n,A,\sigma ,t_{f}\right)\times t_{f}=\frac{nft_{f}(A-vt_{f})}{vT}{\left(1-\frac{A+vt_{f}}{vT\sigma }\right)}^{n-1}$$

**Figure 2:**
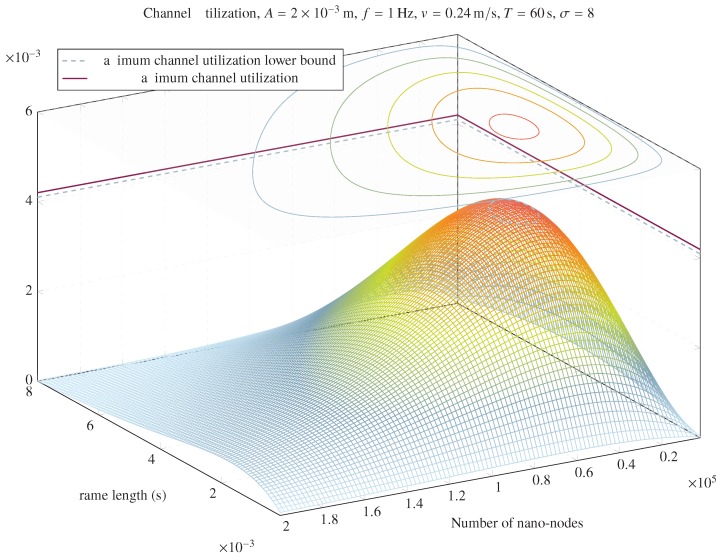
Channel utilization as a function of *n* and $t_{f}$.

From Equation (32), it is easy to derive the value of $t_{f}$, $t_{fU}$, which maximizes $U(n,A,\sigma ,t_{f})$. An upper bound can be obtained considering the following (as shown in Figure 2):

(33) $$U\left(n,A,\sigma ,t_{f}\right)\leq \frac{nft_{f}(A-vt_{f})}{vT}\Rightarrow t_{fU}\leq \frac{A}{2v}$$

Equations (28) and (33) allow us to define a lower bound of the maximum achievable throughput expressed as channel utilization, as follows:

(34) $$\max U\left(n,A,\sigma ,t_{f}\right)\geq \frac{A\sigma }{6ve}$$

Figure 2 represents an example of channel utilization as a function of the frame length ($t_{f}$) and the number of nano-nodes, with fixed values of *A*, *f*, *v*, *T*, and σ. It can be seen that there is a number of nano-nodes and a frame length that maximize channel utilization. Moreover, it can be observed that the lower bound of the maximum of the channel utilization obtained in Equation (33) is close to its real maximum.

## 4. Results

The analytical model in Section 3 was validated by means of simulations. Network parameters for these simulations are based on those of [7], where the authors modeled a flow-guided nano-network inside the human body. Table 2 specifies these values, including the number of slots used in the simulation.

The aim of these simulations is not to build a very detailed nano-network simulator at the link or physical layers, which is a future goal, but to develop a rigorous transmission model abstracting the following main features:

- Nano-nodes are randomly deployed in a circulatory system of length vT.
- There is only one nano-router in the network, although results can proportionally be generalized to more nano-routers, as they are independent.
- The nano-router coverage area is of length *A*.
- Only a single transmission within the coverage of the nano-router is considered to be a successful frame transmission. No frames are received by the nano-router otherwise.
- Two or more simultaneous transmissions within the coverage area always collide.
- Nano-nodes can only send a frame between two battery charges.
- Nano-nodes randomly choose a transmission slot at every transmission cycle.
- Nano-nodes move at an average speed *v* in the flow.

This model has been programmed in Matlab, and the simulation scenario variables are the number of nano-nodes, the number of slots, the length of the coverage area, the battery charging frequency, the nano-node speed in the flow, the time to complete a round in the flow, and the simulation seed. Every point in the simulations is repeated 10 times with a different seed.

### 4.1. Throughput Validation

**Figure 3:**
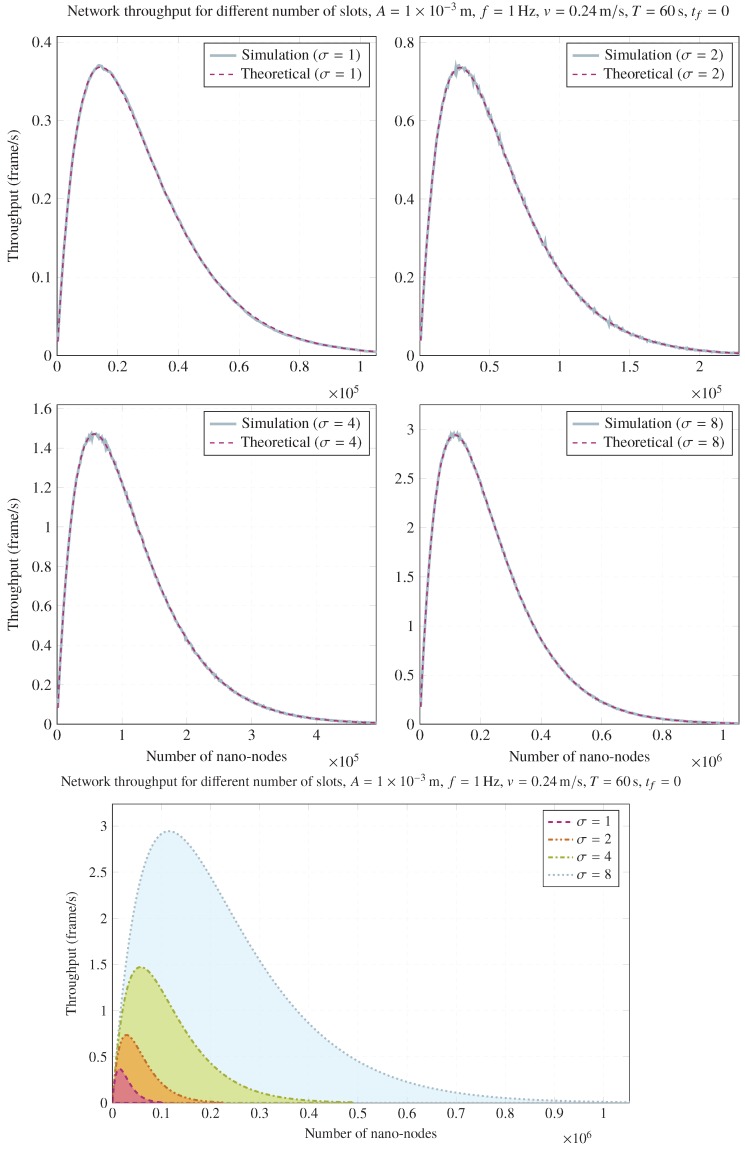
Impact of number of slots on network throughput for $A=1\times 10^{-3}\;m$, f=1Hz, v=0.24m/s, T=60s, and $t_{f}=0$.

Figure 3 illustrates a comparison of network throughput using different numbers of slots for every scenario. These simulations assume that $t_{f}\approx 0$. As can be observed, simulations match with the throughput predicted by the analytical model. These plots show that a greater number of slots allows us to reach a larger throughput, but a higher number of nano-nodes is required. In fact, a larger number of nano-nodes without increasing the number of slots would only worsen the achieved throughput, or a greater number of slots without raising the number of nano-nodes would hardly have any effect for any node within the stability zone (i.e., when the collision probability can be still neglected or the number of nano-nodes is not too high).

In the lower part of Figure 3, it can easily be seen that increasing the number of slots allows for a proportional growth of the maximum achievable throughput at the cost of also increasing the number of nano-nodes. This was previously predicted by Equations (10) (the number of nano-nodes that maximize throughput) and (14) (the maximum achievable throughput).

As can be seen, enlarging the number of slots and the number of nano-nodes is the only way to increase the maximum achievable throughput. It might be possible that the number of nano-nodes is fixed; consequently, independently of the number of slots, the maximum throughput will be upper bounded by nAf/vT, in accordance with Equation (8). This happens when σ→∞; i.e., for an arbitrary number of nano-nodes, the maximum throughput is achieved when σ→∞. Consequently, in any flow-guided nano-network setup, the largest σ possible should be used. The calculation of this value is beyond the scope of this paper, but it depends on the maximum battery charging frequency and the frame length.

**Figure 4:**
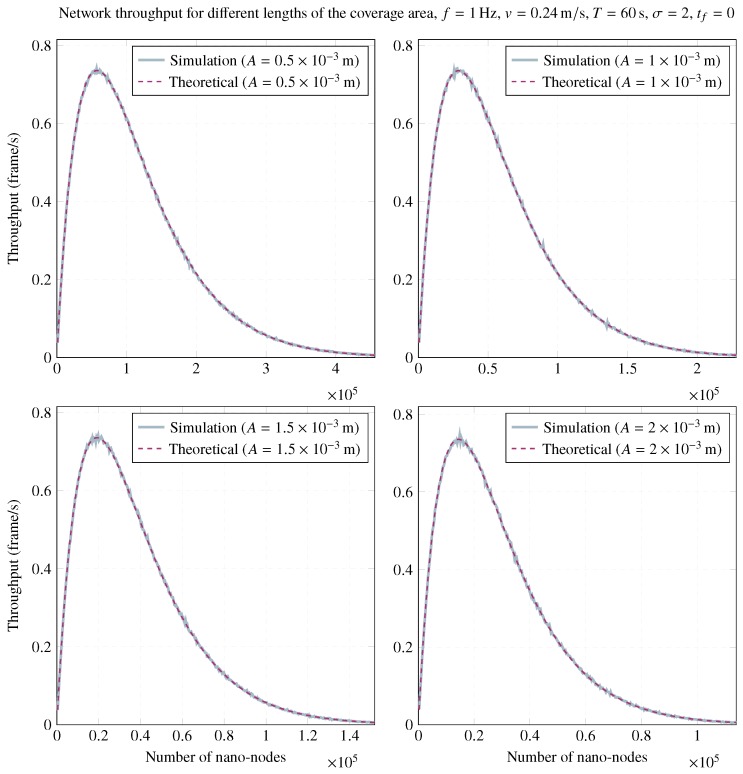

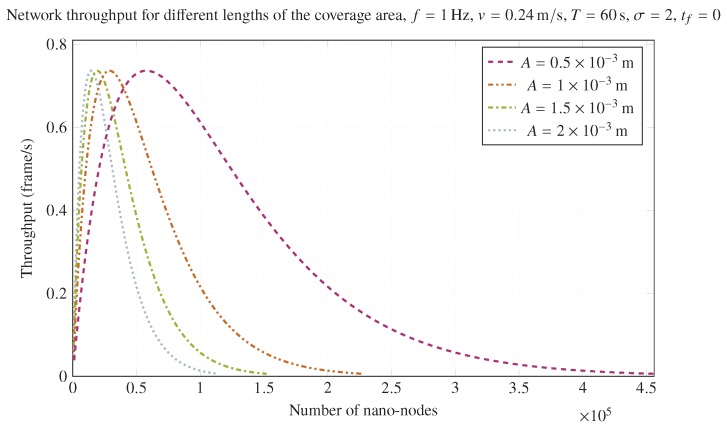
Influence of the length of the coverage area on network throughput for f=1Hz, v=0.24m/s, T=60s, σ=2, and $t_{f}=0$.

Regarding the length of the coverage area, *A*, Figure 4 plots the simulation results for the achieved throughput using different lengths of the coverage area, maintaining the remaining parameters constant. These simulations also validate the outcomes predicted by theory, and, as can be observed, the length of the coverage area has no significant influence on the maximum achievable throughput. However, lower lengths of the coverage area require more nano-nodes to achieve maximum throughput, as was unveiled by Equations (10) and (11). This can be more easily observed in the lower part of Figure 4.

The intuition behind these findings is that, by choosing larger lengths of the coverage area, a better solution for this type of network exists since fewer numbers of nano-nodes are required to achieve maximum throughput. However, albeit outside the scope of this paper, other parameters must be taken into consideration, such as the network lifetime and the average nano-node lifespan set in the network design. In order to configure a flow-guided nano-network with a given lifetime, it may be necessary to use a larger number of nano-nodes, which as theory and simulations indicate, could lead to poor throughputs. To avoid this, a possible solution could be the use of lower transmission powers in the nano-nodes to reduce the length of their coverage range (*A*) and, as nano-nodes start dying, increase it to maximize throughput during the network lifetime.

**Figure 5:**
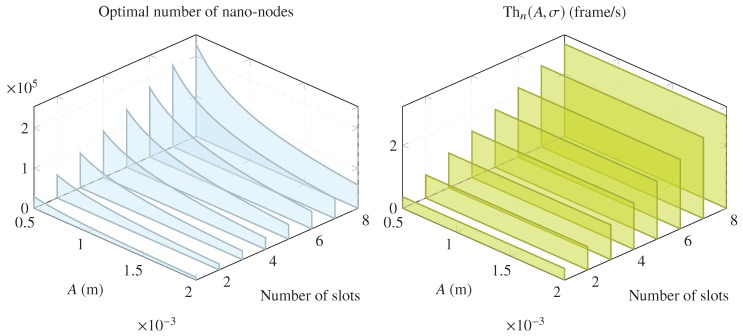
Relation between the optimal number of nano-nodes and the maximum achievable network throughput for f=1Hz, v=0.24m/s, T=60s, and $t_{f}=0$.

Figure 5 represents the relationship between the length of the coverage area, the number of slots, the number of nano-nodes, and the nano-network throughput. Increasing or decreasing *A* causes more or fewer nano-nodes to be required to achieve maximum throughput. Moreover, increasing the number of slots allows us to achieve higher network throughput at the expense of proportionally increasing the number of nano-nodes. As mentioned above, Figure 5 shows that the length of the coverage area is not significant to achieve maximum throughput. To this end, a proper setup of the required amount of nano-nodes and the number of slots is sufficient.

**Figure 6:**
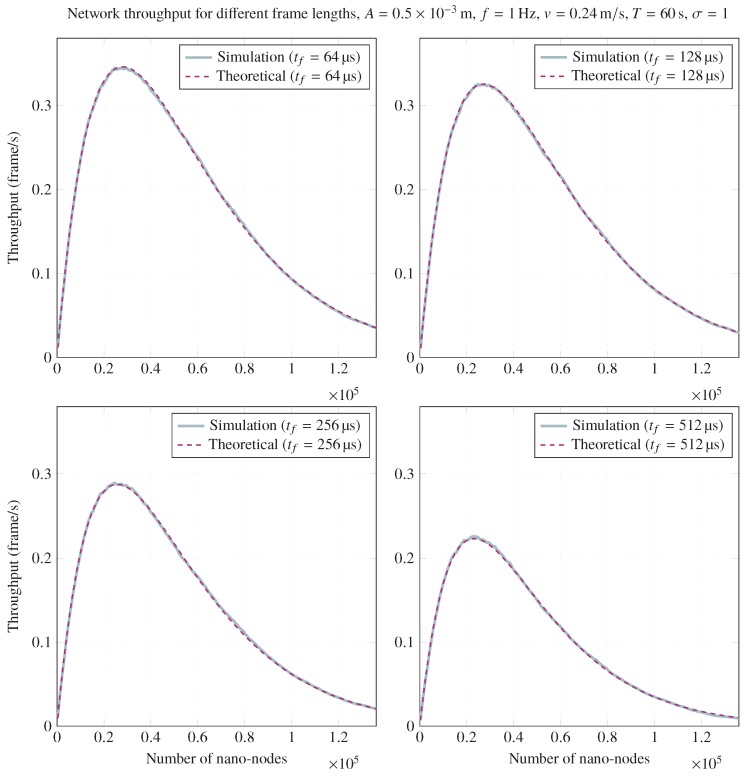

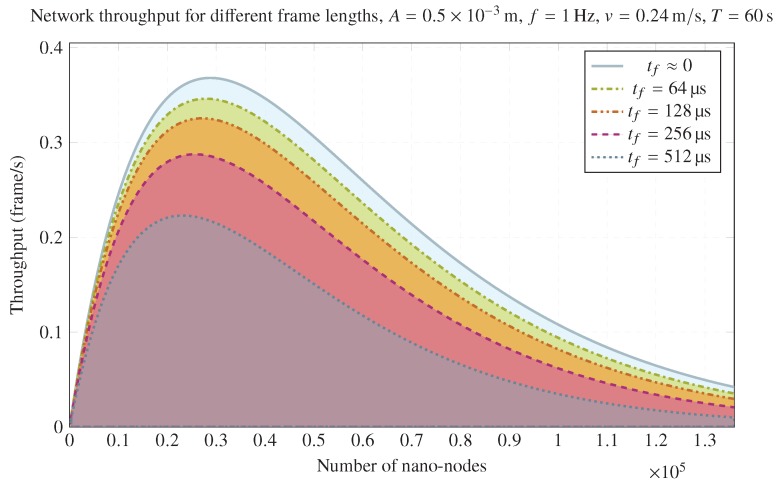
Network throughput for different frame lengths for $A=0.5\times 10^{-3}\;m$, f=1Hz, v=0.24m/s, T=60s, and σ=1.

**Figure 7:**
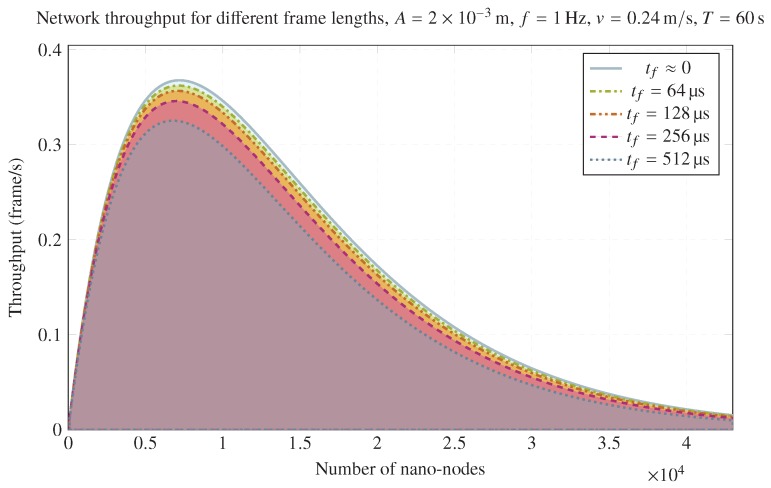
Network throughput for different frame lengths for $A=2\times 10^{-3}\;m$, f=1Hz, v=0.24m/s, T=60s, and σ=1.

Finally, frame length impact on throughput can be observed in Figure 6. It illustrates the achieved throughput for the same scenario for different frame lengths. As can be expected, a longer frame length reduces network throughput as a consequence of increasing the collision area and decreasing the transmission area. However, frame length should be neglected in most cases; if $A≳vt_{f}$, network throughput reduction can be significant. To this end, larger values of *A* can provide better network throughput values, as can be seen in the lower part of Figure 6 and Figure 7.

### 4.2. Collision Rate Validation

**Figure 8:**
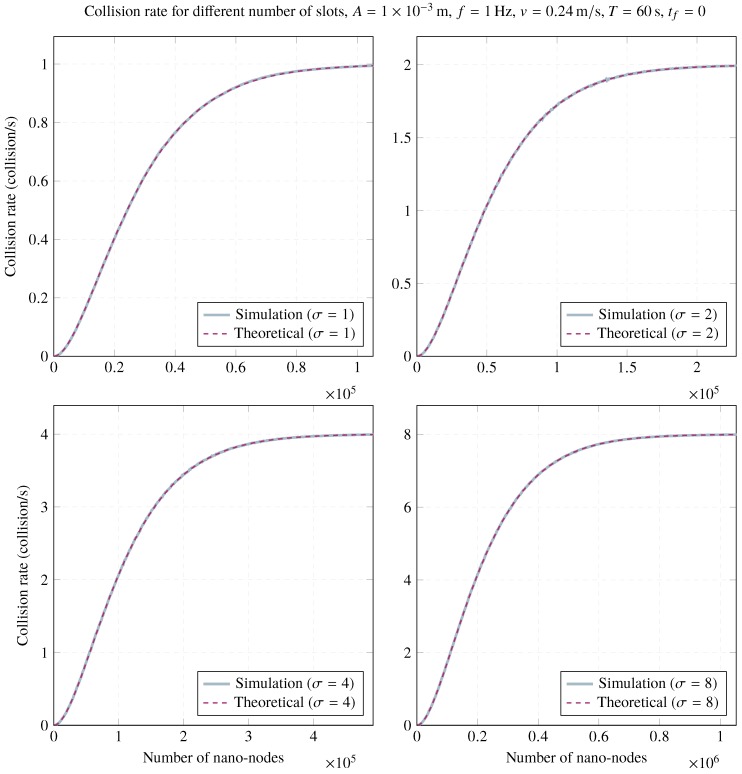
Impact of slots on the network collision rate for $A=1\times 10^{-3}\;m$, f=1Hz, v=0.24m/s, T=60s, and $t_{f}=0$.

The frame collision rate has also been validated by means of computer simulations. Figure 8 depicts the behavior of the collision rate as the number of nano-nodes grows for different numbers of slots, keeping the rest of the parameters constant. Simulations also validate results provided by the analytical model. As can be seen, in all cases the collision rate converges to fσ as the number of nano-nodes escalates. In other words, a frame collision occurs with high probability for any transmission attempt (in any time slot) as the number of nano-nodes increases.

**Figure 9:**
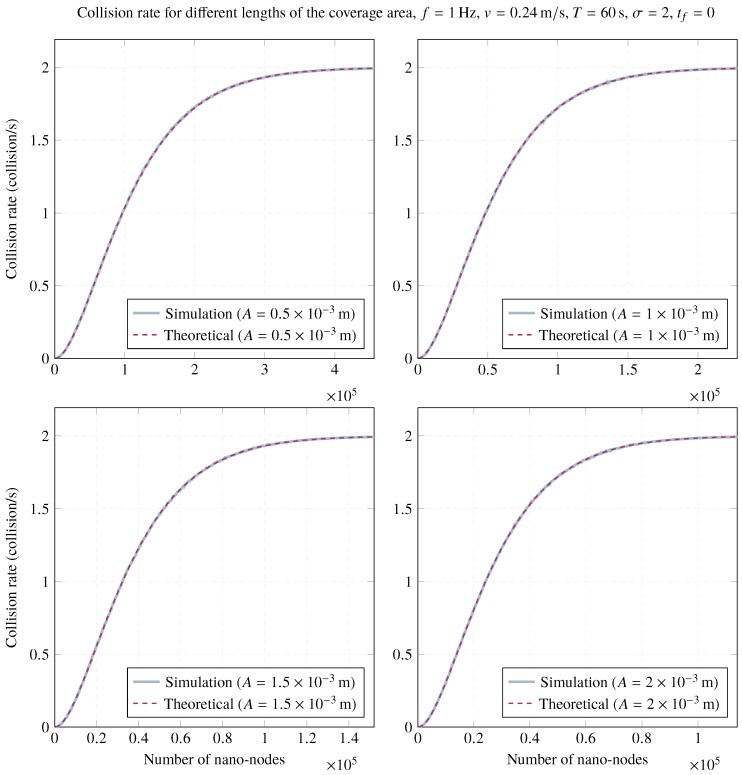
Impact of the length of the coverage area on the network collision rate for $A=1\times 10^{-3}\;m$, f=1Hz, v=0.24m/s, T=60s, σ=2, and $t_{f}=0$.

Regarding the influence of varying the length of the coverage area on the collision rate, Figure 9 shows several simulation results for different lengths of the coverage area. Again, simulation results validate those obtained by the analytical model. As shown in Figure 9, lower lengths of the coverage area reduce the collision rate for a lower number of nano-nodes. However, all studied scenarios converge to the maximum collision rate as the number of nano-nodes increases. Therefore, if frame collision reduction is the main design concern, it might be interesting to reduce *A*, although less throughput will be achieved.

## 5. Conclusions and Future Directions

This paper establishes a theoretical framework to analyze flow-guided nanocommunication networks. It is further validated by means of computer simulations. The mathematical model presented enables the evaluation of the main metrics of flow-guided nano-networks, such as the network throughput or the frame collision rate, as a function of the number of nano-nodes, the coverage range of nano-routers, the battery recharging frequency, the average nano-node velocity, and the number of transmission slots. Results revealed the existence of a limit for the maximum achievable throughput, which depends mainly on the charging frequency of the batteries and the number of available time transmission slots during the charging cycles. In addition, to reach this limit, an optimal number of nano-nodes in the network are required. Below this limit, the addition of more nano-nodes in the network translates into more successful transmissions and, consequently, a throughput increase. Above this limit, the occurrence of collisions becomes more likely, leading to a throughput reduction. This unstable behavior makes throughput tend toward 0, due to the saturation of the channel.

It is also shown that the length of the coverage area has no significant impact on the maximum achievable throughput. However, the optimal number of nano-nodes deployed throughout the network depends on this length. The larger the length of the coverage area is, the fewer nano-nodes are needed to reach the maximum achievable throughput.

Frame length can be neglected if the length of the coverage area is large enough. However, a combination of long frames and a reduced coverage area may have a significant impact on reducing the maximum achievable throughput. Moreover, frame length is important in order to maximize channel utilization. Thus, a proper frame length and the right number of nano-nodes can maximize channel utilization.

Regarding the number of slots, only increasing their number does not imply a substantial improvement in network performance. To have a noticeable effect on network throughput, it is necessary to increase the number of nano-nodes in the same proportion.

Simulations presented in this paper should be observed as a first approach to validate our analytical model. In this sense, we are currently developing a simulation framework based on BitSimulator, a wireless nanonetwork simulator [21,22] that is designed to gracefully scale and support millions of nano-nodes. BitSimulator enables the exploration and understanding of the effects of low-level coding and channel access contention. Our aim is to work with simulation models that consider issues that are difficult to model and thus have not been included in the analytical model.

This work represents a step forward in the effort to develop innovative (realistic) applications based on flow-guided nano-networks. It should also be taken as a basis to enable the implementation of feasible protocols for these networks.

## Figures and Tables

**Table 1:** Variables used in the analytical model and their description.

Variable	Description
*n*	The number of nano-nodes, uniformly distributed, in the flow-guided nano-network, n≥1, n∈N.
*v*	Average speed of the nano-node in the flow in m/s.
*T*	Time required by a nano-node to complete a round in the flow-guided network in s.
*f*	Nano-node charging frequency in Hz.
*A*	Length of the coverage area in m.
$A_{tx}$	Length of the transmission zone in m, $A_{tx}=A-vt_{f}$.
$A_{cx}$	Length of the collision zone in m, $A_{cx}=A+vt_{f}$.
σ	Number of transmission slots between two battery charges, σ≥1, σ∈N.
$t_{f}$	Time required to transmit a frame in s.

**Table 2:** Values of the model parameters used in the simulations.

Variable	Range of Values
*v*	0.24m/s
*T*	60s
*f*	1Hz
*A*	0.5–$2\times 10^{-3}\;m$
σ	1, 2, 4 and 8
$t_{f}$	0–512μs

**Figure A1:**
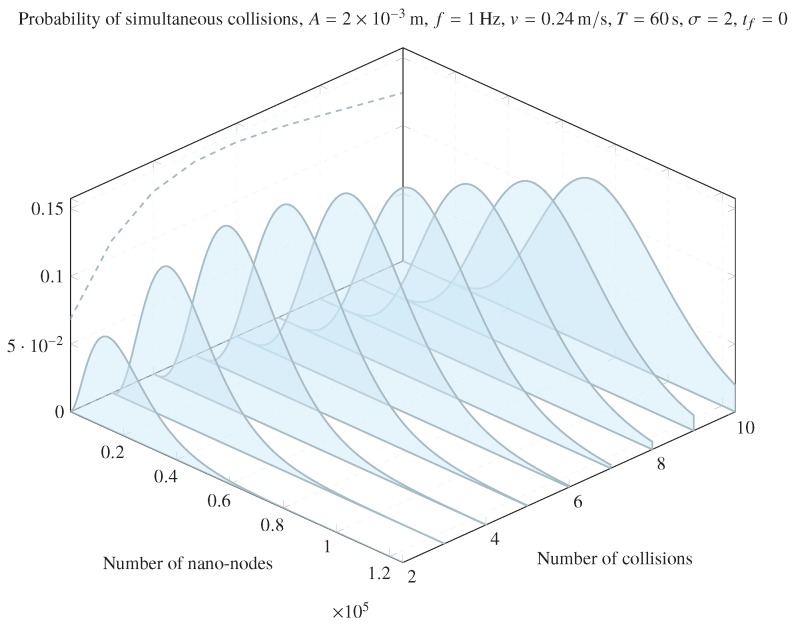
Probability of frame collisions for $A=2\times 10^{-3}\;m$, f=1Hz, σ=2, and $t_{f}=0$.

**Figure A2:**
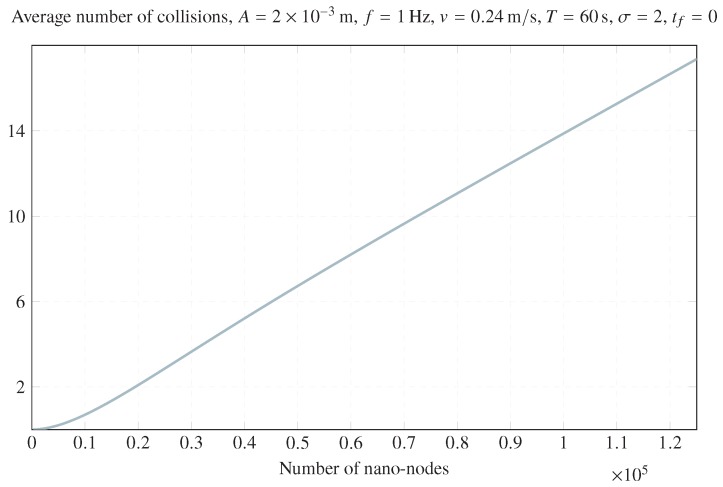
Average number of collisions for $A=2\times 10^{-3}\;m$, f=1Hz, σ=2, and $t_{f}=0$.

## Appendix A. The Average Number of Colliding Transmissions

Section 3.3 offers an analytical equation to calculate the frame collision rate in a flow-guided nanocommunication network, Col(n,A,σ). Let us define $p_{col,k}$ as the probability of *k* nano-node transmissions colliding in the coverage area, k≥2. From Equation (29), $p_{col,k}$ can be expressed as follows:

(A1) pcol,k=(nk)pAk(1−pA)n−k(∑m=2k(km)1σm(1−1σ)k−m)

Equation (A1) can be further simplified to the following:

(A2) pcol,k=(nk)(AvT)k(1−AvT)n−k(1−kσ(1−1σ)k−1−(1−1σ)k)

Figure A1 depicts an example of the probability of simultaneous transmissions (i.e., collisions) as a function of *n*. As can be expected, frame collisions grow with *n*.

From Equation (A1), it is possible to obtain the average number of collisions as a function of *n*:

(A3) E[pcol,k]=∑k=2nk(nk)pAk(1−pA)n−k(∑m=2k(km)1σm(1−1σ)k−m)

Equation (A3) can be simplified as follows:

(A4) $$E[p_{col,k}]=\frac{nA}{vT}(1-{\left(1-\frac{A}{vT\sigma }\right)}^{n-1}(1-\frac{1}{\sigma })-\frac{1}{\sigma }{\left(1-\frac{A}{vT\sigma }\right)}^{n-2})$$

If *n* is large enough, n→∞, $E[p_{col,k}]$ can be approximated by the following:

(A5) $$E[p_{col,k}]\approx \frac{nA}{vT},\;n\to \infty$$

Figure A2 illustrates the behavior of the average number of collisions in a transmission slot. If *n* is large enough, $E[p_{col,k}]$ can be approximated by a linear function of slope nA/vT.
